# Preoperative Gastric Volume Estimation in Diabetic vs Nondiabetic Patients Undergoing Elective Surgeries: A Prospective Observational Study

**DOI:** 10.7759/cureus.101217

**Published:** 2026-01-10

**Authors:** Nimisha Cherunghattil, Rashmi Dubey, Monica Khetarpal, Debajyoti Mohanty, Saroj K Pati

**Affiliations:** 1 Anesthesiology, All India Institute of Medical Sciences, Raipur, IND; 2 Anaesthesiology, All India Institute of Medical Sciences, Raipur, IND; 3 General Surgery, All India Institute of Medical Sciences, Raipur, IND; 4 Radiodiagnosis, All India Institute of Medical Sciences, Raipur, IND

**Keywords:** aspiration, delayed gastric emptying, diabetes, gastric volume, preoperative fasting, ultrasound

## Abstract

Background and aims

Diabetic autonomic neuropathy can cause gastroparesis and delayed gastric emptying. Hence, it can increase the risk of aspiration during induction of anaesthesia, even after adequate fasting and increase morbidity. This study aims to estimate residual gastric volume using ultrasound in diabetic and nondiabetic patients after a period of fasting and oral fluid intake. Additionally, it seeks to evaluate the impact of long-term blood sugar control on gastric emptying.

Methods

This prospective observational study included a total of 80 patients, of which 40 were diabetic patients with a history of diabetes for more than five years, 40 were nondiabetic, all were between 35 to 70 years, American Society of Anesthesiologists (ASA) physical status of I-III, scheduled for non-GI elective surgery under spinal anesthesia. Gastric ultrasound was performed after 8 hours of fasting, and then 2 hours after the intake of 200 ml of plain water orally. Craniocaudal (CC) and anteroposterior (AP) were measured, and the antral cross-sectional area (CSA) and gastric volume were calculated. Additionally, the study assessed the impact of diabetes duration, HbA1C levels, and random blood sugar (RBS) on gastric emptying in the diabetic group. For continuously distributed data independent sample t-test was used, and for non-normally distributed data, the Wilcoxon Test/Kruskal-Wallis test was used for the comparisons. Chi-squared test was used for group comparisons for categorical data. Statistical significance was kept at p < 0.05.

Results

The calculated CSA and GV in diabetics after 8 hours of fasting were 6.91 ± 1.98 cm^2^ and 55.61± 25.65 ml, respectively, which were significantly higher than in non-diabetics, 4.33 ± 0.96 cm^2^ and 20.43 ± 10.37 ml (p < 0.001). Similarly, the calculated CSA and GV in diabetics after 2 hours of fluid intake were 7.50 ± 2.07 cm^2^ and 64.30 ± 27.10 ml, respectively, which were significantly higher than in non-diabetics, 4.61 ± 1.01 cm^2^ and 24.46 ± 11.28 ml (p < 0.001). Additionally, a strong positive correlation was found between HbA1c (%) and fasting gastric volume (p < 0.001), with gastric volume increasing by 16.12 mL for each 1% increase in HbA1c. Similarly, duration of diabetes and Random blood sugar levels also showed a strong positive correlation with gastric volume, with an increase of 6.21 mL per year of diabetes and an increase of 0.66 mL per 1 mg/dL increase in RBS, respectively.

Conclusion

Patients with prolonged duration of diabetes and poor glycemic control have higher residual gastric volume; hence, USG-guided assessment can help to take preventive measures for aspiration preoperatively.

## Introduction

Impairment of airway reflexes and decreased lower oesophageal tone caused by anaesthesia significantly increases the likelihood of gastric content regurgitation and pulmonary aspiration. It can lead to inflammatory response, pneumonitis and eventually to adult respiratory distress syndrome (ARDS) [1-3]. Hence, the American Society of Anesthesiologists (ASA) recommends a minimum of 2 hours of fasting for clear fluids, 6 hours for light meals, and 8 hours for fatty foods to ensure an ‘empty’ stomach at the time of anaesthetic induction [4].

Chronic diseases like diabetes and renal diseases can cause gastropathy due to autonomic dysfunction, hence delaying gastric emptying, especially in poor control [5]. It can increase the risk of aspiration even after the recommended fasting periods. Still, we are following the same fasting guidelines for diabetic patients as for the general population, with no consensus on the appropriate fasting duration for this group [6].

Portable ultrasound (USG) can quickly evaluate gastric contents and stratify risk by distinguishing between empty, clear, or solid volumes [6-9]. Hence, pre-induction ultrasound-guided residual gastric volume estimation will help to assess the risk of aspiration and requirement of preventive measures like rapid sequence induction or extending the duration of fasting, especially in long-duration diabetes with poor control.

Currently, no specific recommendations are available on this concern. Hence, this study was designed to compare residual gastric volume between diabetic and non-diabetic individuals following fixed fasting periods for solids and clear liquids before elective surgery, using USG for real-time evaluation of gastric contents. Additionally, the study explored the impact of long-term blood sugar control on gastric emptying.

## Materials and methods

The study received approval from the Institutional Ethics Committee, and it has been reported as per the STROBE guidelines. Written informed consent was obtained from all participants prior to enrollment. Participants included patients scheduled for non-GI elective surgery under spinal anesthesia. Both male and female patients aged 35 to 70 years, belonging to ASA physical status I-III, were included. For the diabetic cohort, only those with a minimum of five years of diagnosed diabetes mellitus were selected. Patients were excluded if they had sepsis, shock, pregnancy, gastroesophageal reflux disease (GERD), prior upper abdominal or gastrointestinal surgery, BMI > 35 kg/m², or any factor preventing accurate gastric volume assessment.

Participants were divided into two groups: Group A (diabetic) included patients with a history of diabetes mellitus (DM) of at least five years, while Group B (non-diabetic) comprised patients without diabetes. The primary objective was to compare residual gastric volume between diabetic and non-diabetic patients after 8 hours of fasting. Secondary objectives included: (1) assessing residual gastric volume 2 hours after administration of 200 mL of plain water and comparing the results between groups, (2) evaluating the relationship between glycosylated hemoglobin (HbA1c) levels and gastric emptying in the diabetic cohort, and (3) examining the association between diabetes duration and gastric emptying.

USG-guided gastric volume estimation was done twice, the first time at 8 hours of fasting and then 2 hours after oral intake of 200 ml of plain water. Patients were scanned in the supine position, then in right lateral decubitus (RLD) position using a low frequency (2-5 MHz), curved array 60 mm transducer with a depth of up to 30 cm on a Sonosite ultrasound system.

The gastric antrum, the most accessible part of the stomach for sonographic imaging, was evaluated in a sagittal or parasagittal epigastric plane, positioned between the left liver lobe (anteriorly) and the pancreas (posteriorly). The aorta, inferior vena cava (IVC), and superior mesenteric vessels served as anatomical landmarks to ensure consistent scanning.

Cross-sectional area (CSA) was calculated by using two perpendicular diameters - anteroposterior (AP) and craniocaudal (CC) and the formula for the area of an ellipse [10]:

${CSA} = \frac{AP \times CC \times \pi}{4}$

The gastric volume was calculated using the formula, which was previously validated [11,12]:

$\text{Volume (mL)} = 27 + (14.6 \times \mathrm{CSA}_{\mathrm{right}}) - (1.28 \times \mathrm{Age})$

Gastric volume was estimated using the Perlas-derived formula [11,12], which was originally validated in healthy adult volunteers and general surgical populations rather than specifically in patients with diabetes.

Glycosylated hemoglobin can be used to assess the blood sugar control of the patient in the last 90 days, as the RBC life span is 90 days. Hence, we can assess the impact of blood sugar control on gastric emptying by evaluating the relationship between HbA1C and gastric volume. HbA1C was graded into three groups as normal (< 5.7%), prediabetic (5.7 - 6.4%) and diabetic (>6.5) and GV was compared in these three groups. The HbA1c subgroup analysis was performed exclusively within the diabetic group for correlation analysis.

The sample size was calculated based on the primary outcome of the difference in mean gastric antral cross-sectional area (CSA) between diabetic and non-diabetic patients after fasting. Estimates were derived from the study by Garg et al. [6], which reported mean CSA values of 2.30 ± 1.18 in non-diabetic patients and 3.73 ± 1.61 in diabetic patients. The pooled standard deviation was 1.41, and the expected mean difference was 1.43. Sample size estimation was performed using the formula described by Snedecor and Cochran (1989) [13], assuming a two-sided alpha error of 5% and a power of 99%. The calculated sample size was 40 participants per group (total = 80).

Data were entered into Microsoft Excel and analysed using SPSS version 23 (IBM Corp.). Descriptive statistics were expressed as mean ± standard deviation (SD) or median with interquartile range (IQR) for continuous variables, and as frequency and percentage for categorical data. Graphical representations such as histograms, box-and-whisker plots, and bar charts were used for visualisation. Group comparisons of continuous variables were performed using the independent t-test for two groups and one-way ANOVA for comparisons involving more than two groups. Tukey’s HSD test was applied post-hoc to adjust for multiple comparisons. For non-normally distributed data, Wilcoxon or Kruskal-Wallis tests were used. Categorical variables were analysed using the Chi-square test, and when expected frequencies were <5 in >25% of cells, the Fisher’s exact test was applied. Correlations between continuous variables were assessed using Pearson’s or Spearman’s correlation coefficients, depending on normality. Statistical significance was considered at p < 0.05.

## Results

A total of 80 patients were included, with 40 in each group. Both groups were comparable demographically in terms of age, gender distribution and BMI (Table 1). There was a significant difference found between the groups in terms of distribution of ASA Class (p = <0.001). About 72.5% of the participants in group A had ASA Class: II, and the remaining 27.5% of the participants in group A had ASA Class: III. 70.0% of the participants in group B had ASA Class: I. 30.0% of the participants in group B had ASA Class: II. Participants had a mean diabetes duration of 7.55 years, and the interquartile range was between 5 and 10 years.

**Table 1:** Demographic profile of both the groups. ***Significant at p<0.05, 1: Continuous variables, reported as mean ± SD, Statistical test used is t-test, 2: Categorical variables, reported as N (%), Statistical test used is Chi-Squared Test

Parameters	Group		Test statistic value	p value
	Diabetic (n = 40)	Nondiabetic (n = 40)		
Age (Years)	56.45 ± 8.34	54.52 ± 7.71	t = 1.072^1^	0.287
Gender				
Male	24 (60.0%)	23 (57.5%)	χ2 = 0.052^2^	0.820
Female	16 (40.0%)	17 (42.5%)		
BMI (Kg/m²)	22.56 ± 2.08	21.89 ± 1.76	t = 1.555^1^	0.124
ASA Class***				
I	0 (0.0%)	28 (70.0%)	χ2 = 46.049^2^	<0.001
II	29 (72.5%)	12 (30.0%)		
III	11 (27.5%)	0 (0.0%)		

**Figure 1:**
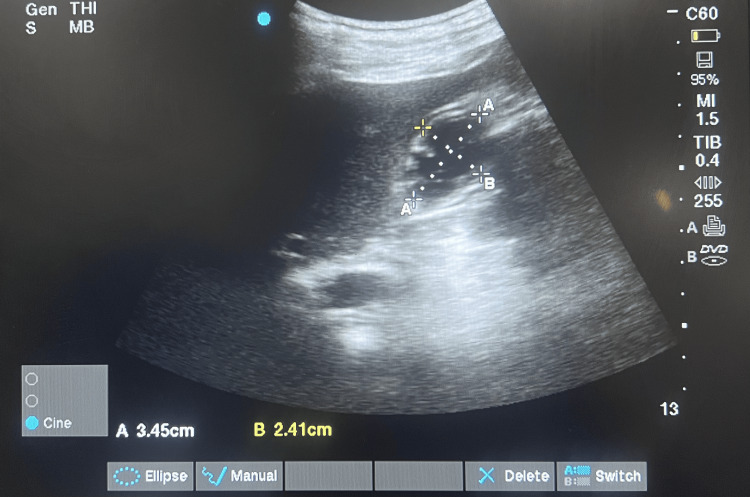
USG image of Gastric antrum AP (A) and CC (B) diameters (cm)

The measurement of AP and CC diameters using a portable ultrasound machine is depicted in Figure 1. The measurement of AP and CC diameters and CSA and GV is depicted in Table 2 (fasting) and Table 3 (post oral fluid intake).

**Table 2:** Comparison of gastric diameters, CSA, and volume in both groups (fasting). ***Significant at p<0.05, 1: Wilcoxon-Mann-Whitney U Test, 2: t-test

Parameters	Group		Test statistic value	p value
	Diabetic (n = 40)	Nondiabetic (n = 40)		
Gastric Antrum AP Diameter (cm) (Fasting)***	3.40 ± 0.48	2.78 ± 0.53	w = 1290.50^1^	<0.001
Gastric Antrum CC Diameter (cm) (Fasting)***	2.56 ± 0.50	1.99 ± 0.29	t = 6.235^2^	<0.001
Gastric Antrum CSA (cm) (Fasting)***	6.91 ± 1.98	4.33 ± 0.96	t = 7.410^2^	<0.001
Gastric Volume (mL) (Fasting)***	55.61 ± 25.65	20.43 ± 10.37	t = 8.042^2^	<0.001

**Table 3:** Comparison of gastric diameters, CSA, and volume in both groups (post-oral-fluid). ***Significant at p<0.05, 1: t-test; CSA: Cross-sectional area.

Parameters	Group		Test statistic value	p value
	Diabetic (n = 40)	Nondiabetic (n = 40)		
Gastric Antrum AP Diameter (cm) (Post-Oral-Fluid) ***	3.65 ± 0.50	2.89 ± 0.51	t = 6.704^1^	<0.001
Gastric Antrum CC Diameter (cm) (Post-Oral-Fluid) ***	2.58 ± 0.46	2.03 ± 0.31	t = 6.282^1^	<0.001
Gastric Antrum CSA (cm) (Post-Oral-Fluid) ***	7.50 ± 2.07	4.61 ± 1.01	t = 7.960^1^	<0.001
Gastric Volume (mL) (Post-Oral-Fluid) ***	64.30 ± 27.10	24.46 ± 11.28	t = 8.585^1^	<0.001

The fasting mean AP and CC diameters of diabetics were 3.40 ± 0.48 cm and 2.56 ± 0.50 cm, and in non-diabetics were 2.78 ± 0.53 cm and 1.99 ± 0.29 cm, respectively. The calculated CSA and GV in diabetics after 8 hours of fasting were 6.91 ± 1.98 cm^2^ and 55.61± 25.65 ml, respectively, which were significantly higher than in non-diabetics, 4.33 ± 0.96 cm2 and 20.43 ± 10.37 ml (p < 0.001). Similarly, the mean AP and CC diameters of diabetics after 2 hours of fluid intake were 3.65 ± 0.50 cm and 2.58 ± 0.46 cm and in non-diabetics were 2.89 ± 0.51 cm and 2.03 ± 0.31 cm, respectively. The calculated CSA and GV in diabetics after 2 hours of fluid intake were 7.50 ± 2.07 cm^2^ and 64.30 ± 27.10 ml, respectively, which were significantly higher than in non-diabetics, 4.61 ± 1.01 cm2 and 24.46 ± 11.28 ml (p < 0.001).

The change in gastric volume from 8 hours of fasting to 2 hours post oral fluid intake was assessed with the same formula. Diabetics had a mean value of 8.70 ± 6.52 ml, and nondiabetics had a mean value of 4.03 ± 3.58 ml. The p-value obtained was less than 0.001 and hence statistically significant.

**Figure 2:**
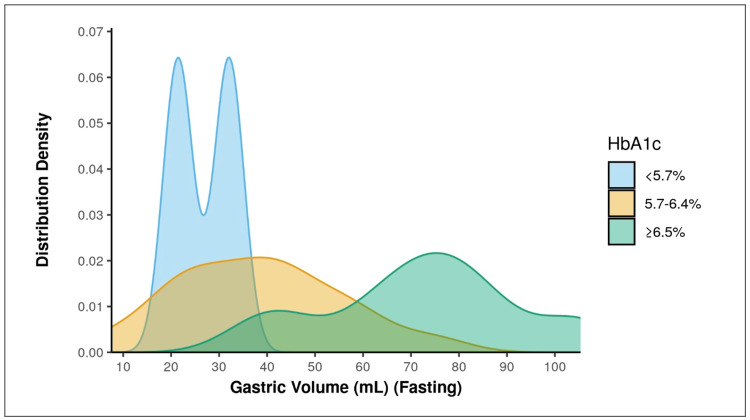
Association between HbA1c and gastric volume (mL) (Fasting)

**Figure 3:**
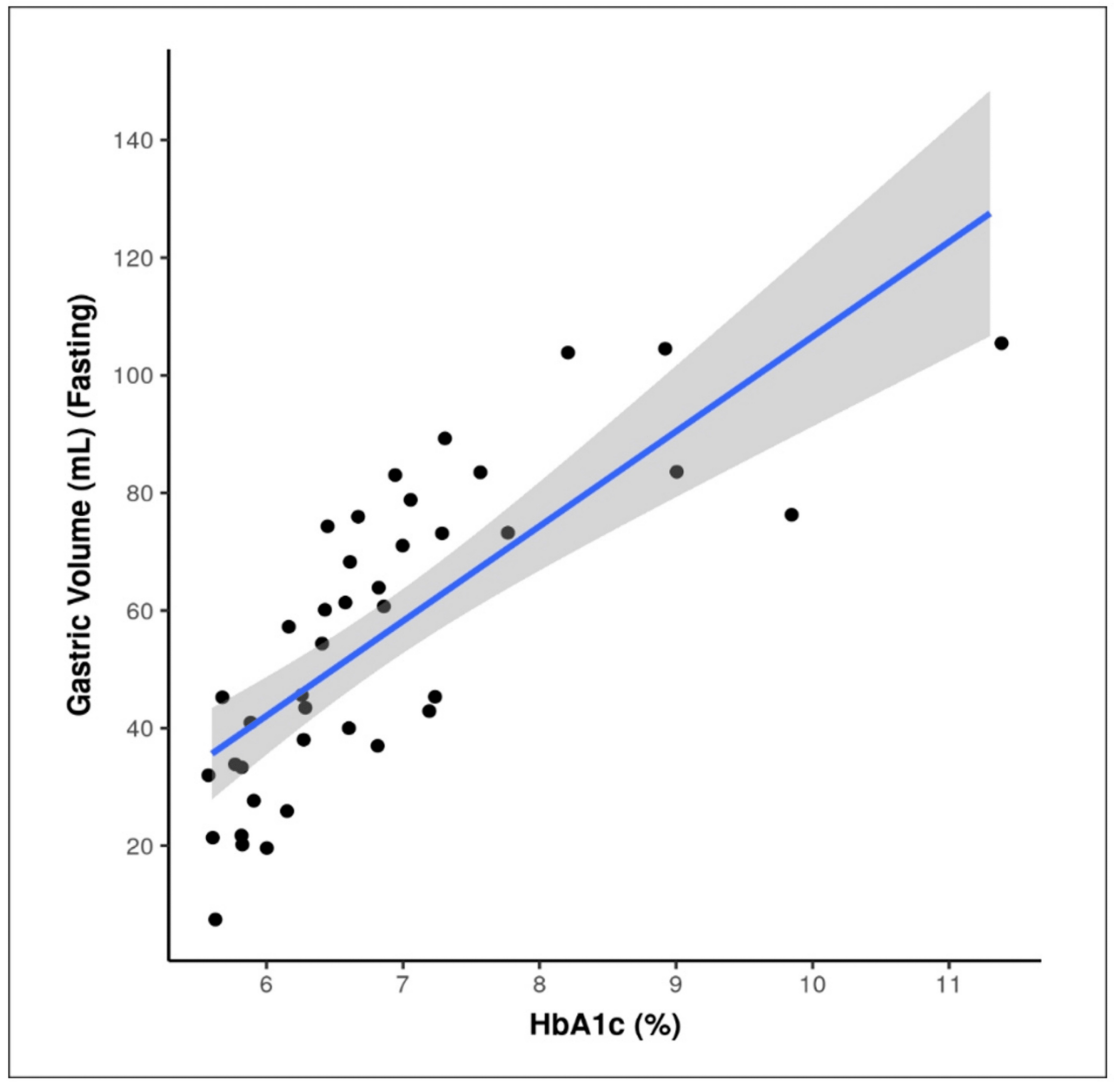
Correlation between HbA1c (%) and gastric volume (mL) (Fasting) Individual points represent individual cases. The blue trendline represents the general trend of correlation between the two variables. The shaded grey area represents the 95% confidence interval of this trendline.

There was a significant difference found between the 3 groups of HbA1c in terms of gastric volume (mL) ((Fasting), p = <0.001)) (Table 4), with being highest in the HbA1c: ≥6.5% group, depicted in Figure 2. So, there is a strong positive correlation between HbA1c and GV, and this correlation was statistically significant as p is <0.001, depicted in Figure 3. GV increases by 16.12 mL for every 1% increment in HbA1c.

**Table 4:** Association between HbA1c and gastric volume (mL) Significant at p<0.05, 1: Kruskal-Wallis Test

Gastric Volume (mL) (Fasting)	HbA1c			Kruskal-Wallis Test	
	<5.7%	5.7-6.4%	≥6.5%	χ2	p value
Mean (SD)	26.75 (7.52)	38.19 (17.23)	72.45 (20.10)	20.1781	<0.001

**Figure 4:**
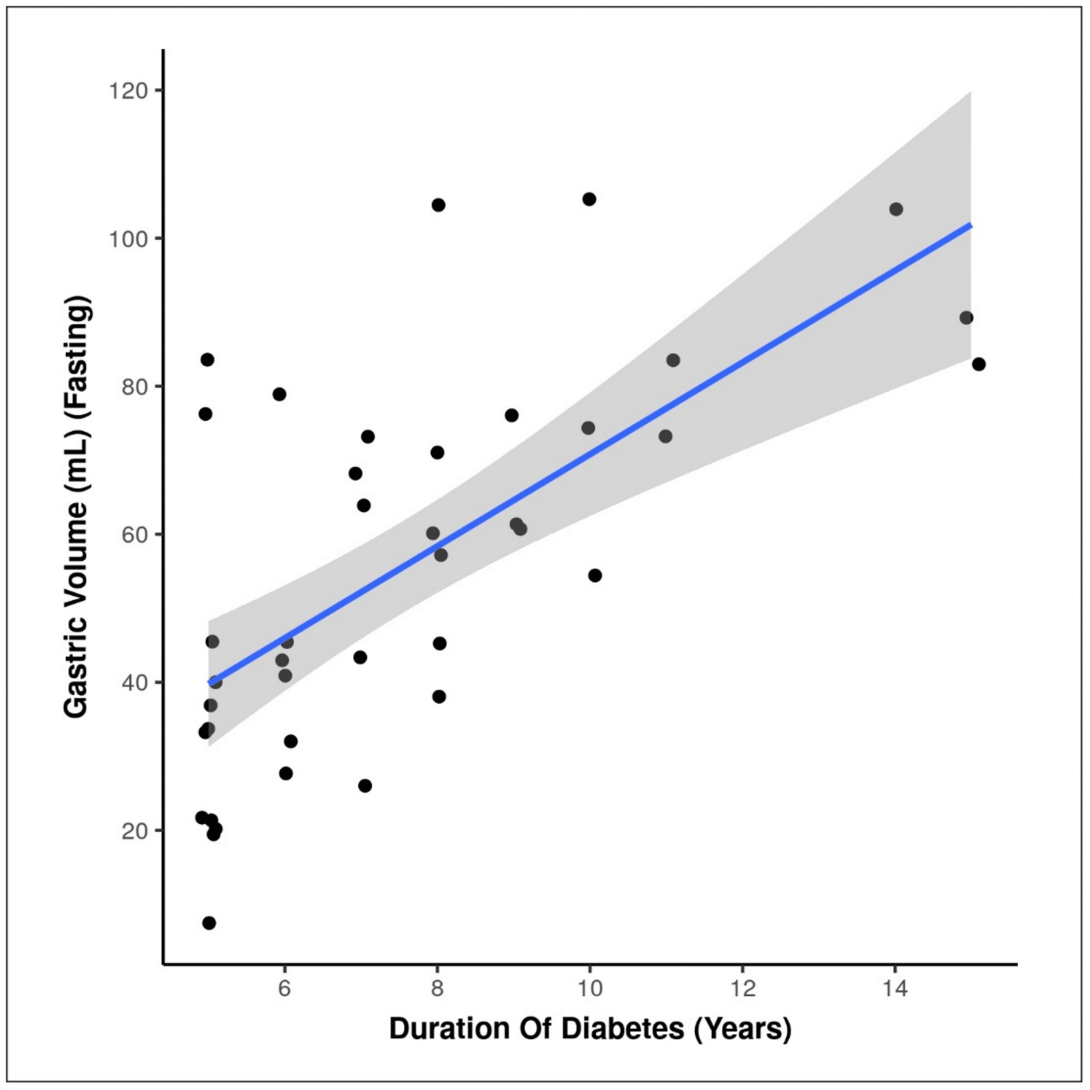
Correlation between duration of diabetes (Years) and gastric volume (mL) (fasting) Individual points represent individual cases. The blue trendline represents the general trend of correlation between the two variables. The shaded grey area represents the 95% confidence interval of this trendline.

And our study was showing there is a strong positive correlation between duration of diabetes (Years) and GV (mL) (fasting), and this correlation was statistically significant (rho = 0.66, p = <0.001). With every 1 unit increase in duration of diabetes (Years), the GV (mL) increases by 6.21 units. Figure 4 scatterplot depicts the correlation between duration of diabetes and GV.

**Figure 5:**
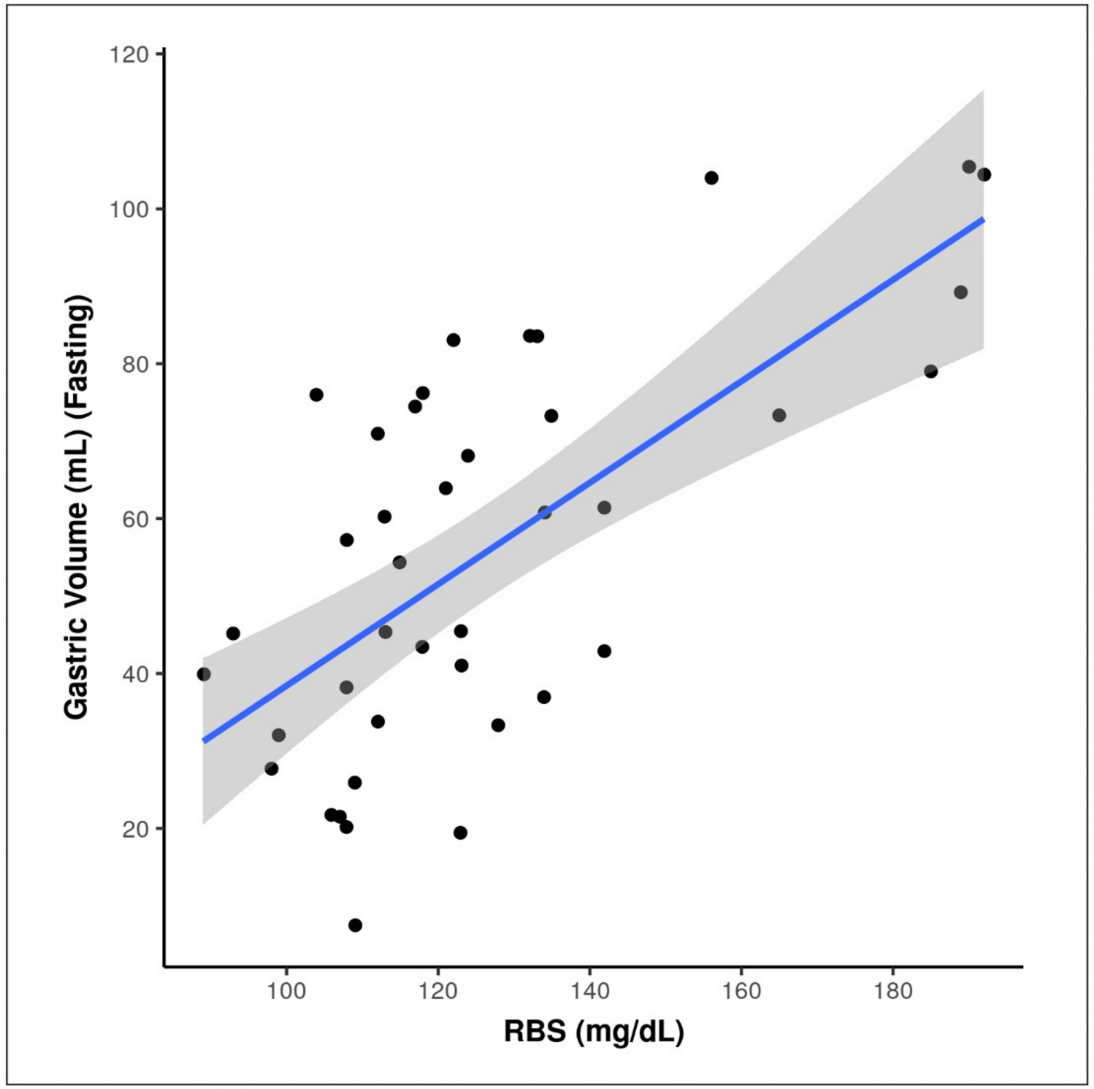
Correlation between RBS (mg/dL) and gastric volume (mL) (Fasting). Individual points represent individual cases. The blue trendline represents the general trend of correlation between the two variables. The shaded grey area represents the 95% confidence interval of this trendline.

There was a strong positive correlation between RBS (mg/dL) and GV (mL) (Fasting), and this correlation was statistically significant (rho = 0.61, p = <0.001). For every 1 unit increase in RBS (mg/dL), the GV (mL) increases by 0.66 units. Figure 5 scatterplot depicts the correlation between RBS (mg/dL) and GV (mL).

## Discussion

Both groups were comparable demographically in terms of age, gender distribution and BMI. There was a significant difference found between the groups in terms of distribution of ASA Class (p = <0.001). It is because group A participants were long-term diabetics with uncontrolled blood sugar values, and group B participants were without any comorbidities. Preoperative baseline vitals were not statistically significant between the two groups. In our study measured gastric dimensions and calculated GV were significantly higher in the diabetic group than nondiabetic group after 8 hours of fasting and after 2 hours of clear liquid intake.

Similar observations were made by Sharma et al. [14] in a comparative observational study assessing ultrasound-guided residual gastric volume among diabetic and non-diabetic patients scheduled for elective procedures under general anesthesia. They reported a mean GV of 15.48 ± 11.18 mL in diabetics, significantly greater than 9.77 ± 18.56 mL in controls (p < 0.0001). The gastric aspirate volume was also higher in diabetics (1.24 ± 1.46 mL) than in non-diabetics (0.3 ± 0.78 mL), demonstrating statistical significance. The authors concluded that patients with long-standing diabetes exhibit increased residual gastric volume and antral CSA despite adhering to standard preoperative fasting guidelines, as determined by ultrasonography.

The American Society of Anesthesiologists (ASA) recommends that 2 hours of fasting for clear liquids preoperatively is adequate for reducing the risk of aspiration, and it will reduce thirst and improve patient satisfaction [4]. Hence, evaluating the residual gastric volume after 2 hours of clear liquid intake will help to modify the existing practice with improvement in patient comfort.

The change in gastric volume from 8 hours of fasting to 2 hours post oral fluid intake was assessed with the same formula. Diabetics had a mean value of 8.70 ± 6.52 ml, which was significantly higher than 4.03 ± 3.58 ml in nondiabetics.

Although CSA and GV values differed significantly between the diabetic and non-diabetic cohorts, both remained within a range considered low for aspiration risk. Although there is no strict volume threshold above which increased risk of aspiration, the residual volume in a healthy fasted individual, up to 1.5 ml/kg (about 100 ml), is believed to be safe [15]. In our study, only 3 patients had gastric volume more than 100 ml after 2 hours of clear fluid intake. This suggests that, despite statistically significant differences, the absolute magnitude of increase in GV may not uniformly represent clinically significant aspiration risk for all diabetic patients. Clinical relevance becomes more apparent when individual risk factors are considered.

And our study was showing that HbA1c (%) and duration of diabetes have a strong positive correlation with GV (ml). For every 1 unit increase in HbA1c (%) and duration of diabetes (Years), the GV (mL) (Fasting) increases by 16.12 units and 6.21 units, respectively. These findings indicate that patients with long-standing diabetes and poor glycemic control may be more likely to have clinically meaningful residual gastric volumes, even after recommended fasting periods.

Residual gastric volume and resulting gastric regurgitations under general anesthesia can also occur in other conditions like inadequate duration of fasting due to urgent clinical situations or due to communication issues, other chronic conditions associated with autonomic neuropathy like advanced liver or renal dysfunction or critically ill patients, and some situations where a proper fasting history cannot be elicited. In these situations, gastric ultrasound, which is a noninvasive, simple technique, can be used to individualise the aspiration risk and to guide anesthetic management [16].

Heena Garg et al. [6] conducted an observational study on 103 patients to compare fasting gastric volumes in diabetic versus non-diabetic individuals using ultrasound before elective surgery. Both qualitative and quantitative assessments of the gastric antrum were performed in supine and right lateral decubitus positions. Their results demonstrated significantly higher CSA and GV in the diabetic group across both positions. They also applied the sonographic grading system defined by Perlas et al. [17], classifying the antrum as Grade 0 (empty), Grade 1 (fluid only in right lateral position), or Grade 2 (fluid present in both supine and right lateral positions).

Rabab Sabry et al. [18] conducted a similar study for evaluating gastric residual volume in fasting diabetic patients using gastric ultrasound, and they also did nasogastric aspiration of gastric volume after induction of anesthesia. The study group was patients with at least six years of diabetes, and the result showed higher gastric volume in the study group by both techniques, and there was a good correlation between calculated residual gastric volume using ultrasound measures and volume of gastric contents aspirated through nasogastric tube.

Ultrasound assessment of gastric residual volume in patients over 60 years of age undergoing gastroscopy under sedation was done by Wang J et al., who found that there is a higher incidence of full stomach in the study group as compared to the young control group [19]. They used the Perlas et al qualitative grading scale for gastric content in ultrasound imaging [17].

While our study demonstrates statistically significant differences in gastric volume between diabetic and non-diabetic patients, the clinical relevance of these differences depends on absolute gastric volume and individual patient risk factors. These findings support the use of gastric ultrasound for individualised aspiration risk assessment, particularly in patients with long-standing, poorly controlled diabetes, rather than as a routine screening tool for all diabetic patients.

There are limitations in our study. It cannot be implemented in obese patients, in patients with difficulty doing abdominal ultrasound or in pregnancy. Ultrasound measurements can be variable with different examiners, and image interpretation requires experience and skill. Other factors which can also affect gastric emptying are not considered in our study (chronic renal or liver disease). A significant imbalance in ASA physical status was observed between study groups, with diabetic patients more frequently classified as ASA II-III. As ASA status reflects systemic disease burden and may affect gastric motility independent of diabetes, it represents a potential confounder. This limitation should be considered when interpreting the results. Future studies incorporating ASA-matched cohorts or multivariate analyses are needed to clarify the independent effect of diabetes on gastric emptying. The study was observational, and investigators were not blinded to group assignment, which may introduce observer bias and limit causal inference.

## Conclusions

Patients with long-standing diabetes and poor glycemic control may exhibit higher residual gastric volumes, suggesting delayed gastric emptying, even after standard preoperative fasting. As all procedures in this study were performed under spinal anesthesia, these findings should not be generalized to aspiration risk during the induction of general anesthesia.

The results are hypothesis-generating, highlighting the need for further studies to evaluate the clinical significance of increased gastric volumes in diabetic patients and to determine whether individualised fasting protocols improve safety. Preoperative gastric ultrasound may be a useful tool for risk stratification and anesthetic planning in selected high-risk patients, though its routine impact on outcomes requires further validation.
